# Keeping People *Down*, *In (Line)* and *Out*: Structural Stigma and Missingness In Health Care

**DOI:** 10.1177/27551938261419417

**Published:** 2026-02-26

**Authors:** David Baruffati, Mhairi Mackenzie, Calum Lindsay, David Ellis, Michelle Major, Catherine O’Donnell, Sharon Simpson, Geoff Wong, Andrea Williamson

**Affiliations:** 1 General Practice and Primary Care, School of Health & Wellbeing, 223860 University of Glasgow, Glasgow, UK; 2 School of Social & Political Sciences, Urban Studies, 150729 University of Glasgow, Glasgow, UK; 3 General Practice and Primary Care, School of Health and Wellbeing, 223860 University of Glasgow, Glasgow, UK; 4 School of Management, 1555 University of Bath, Bath, UK; 5 Homeless Network Scotland, Glasgow, UK; 6 General Practice and Primary Care, School of Health and Wellbeing, University of Glasgow, Glasgow, UK; 7 MRC/CSO Social & Public Health Sciences Unit, School of Health and Wellbeing, 223860 University of Glasgow, Glasgow, UK; 8 Nuffield Department of Primary Care Health Sciences, 228157 University of Oxford, Oxford, UK; 9 General Practice and Primary Care, School of Health and Wellbeing, 223860 University of Glasgow, Glasgow, UK

**Keywords:** stigma, health inequalities, social determinants of health, health care services, access, inclusion health

## Abstract

Recent years have seen the development of conceptualisations of stigma which have moved beyond individual-level analyses towards exploring how stigma operates across multiple levels. While empirical research has examined the impact of stigma across various domains, there remains scant research exploring the lived experience of *structural stigma*. In this article, we examine structural stigma as a driver of multiple missed appointments, or ‘missingness’, in health care. We draw on qualitative data from 61 interviews with health and social care professionals and experts-by-experience of missingness in the United Kingdom, focusing on three stigmatised statuses: people from marginalised racial and ethnic groups, including those in the asylum system; people with mental health conditions; and people experiencing problem substance use. We adapt Link and colleagues’ schema of stigma outcomes—keeping people *down*, *in,* and *away*—to explore how structural stigma shapes access to and experiences of health care. Our findings demonstrate a range of such processes through which barriers to effective engagement occur, and suggest that a focus on structural stigma will benefit policy, practice, and future research in this area.

In this article we draw on qualitative research exploring multiple missed appointments in health care to explore how structural stigma generates inequalities in access to and the quality of health care in the United Kingdom. Missed appointments are the focus of significant media, policy, and research attention.^1^ This discourse has focused predominantly on the detrimental outcomes for services while neglecting those for patients, and has placed responsibility almost solely on patients. This framing has obscured the substantial barriers which many people face in engaging with health care, and the unequal health outcomes to which these can contribute.^2^ We aim to challenge such perspectives by exploring how barriers to accessing health care form an important causal mechanism linking structural stigma^3^ to poor and unequal health outcomes. Such work is important within the UK context. While the National Health Service (NHS) is free at the point of access, it and other public and third-sector services pertinent to the social determinants of health and Social Security safety nets have been significantly weakened by the post-2010 UK governments’ austerity project.^4^

## Stigma

The work of Erving Goffman^5^ catalysed sociological interest in the origins and consequences of stigma. This literature has remained primarily focused on Goffmanian conceptualisations, portraying stigma as residing in ‘attributes’ of stigmatised persons, and placing focus on individual-level attitudes, behaviours, and outcomes. Only relatively recently have theory and research understood stigma and discrimination as “social processes that can only be understood in relation to broader notions of power and domination”.^6–11p.14-11^ Taking this latter approach, Link and Phelan^11^ provide a useful conceptualisation of stigma. They see stigma as consisting of five components: labelling human differences; attributing negative stereotypes onto these persons; ‘othering’ those labelled; status loss and discrimination leading to unequal outcomes, and the wider structure which allows those with social, economic and political power greater control over each of these components.

### Structural Stigma

Link and Phelan's conceptualisation delineates three levels at which these stigma processes occur: *intrapersonal*, *interpersonal,* and *structural*.^11^ *Intrapersonal stigma* acts through the stigmatised person, including through ‘self-stigma’ (internalised stigmatising attitudes) and the agentic actions of individuals in response to interpersonal and/or structural stigma.^12^ *Interpersonal stigma* comprises direct, person-to-person stigma practices occurring between the stigmatised and non-stigmatised. While interpersonal and resultant intrapersonal stigma have been the predominant focus of stigma theory and research, it stands that “all manner of disadvantage can result outside a model in which one person does something bad to another”.^11p.382^.

Reflecting this, there has been a welcome development of theory and empirical research across disability scholarship,^5^ the health inequalities literature,^6,10,11,13–15^ including the health care literature relating to marginalised groups,^16^ and the wider sociological literature^17^ of analyses of *structural stigma*, which consists of macro-social processes that systematically disadvantage those who are stigmatised. The concept extends the concept of ‘systemic’ and ‘institutional racism’,^18^ and denotes “societal-level conditions, cultural norms, and institutional policies [which] constrain the opportunities, resources and wellbeing of the stigmatised”.^14p.2^ We place focus on structural stigma throughout this article given that structural determinants are the root causes of health inequalities and should be the target of any interventions seeking to address them.^19–23^ Existing research exploring structural stigma in relation to health inequalities has broadly taken two methodological approaches: policy analyses, and surveys extrapolating individuals’ attitudes towards those who are stigmatised.^24^ While valuable, we argue that qualitative research is essential in this area to deepen our understanding of how structural stigma processes operate, how they are experienced, and the outcomes they produce across different contexts.

### Stigma Outcomes

Stigma would not exist if it did not serve a function.^17^ for our analysis, we adapt link and colleagues’ schema of stigma outcomes—keeping people down, in, and away.^15,25^ We are further informed by Pinker's identification of stigma as central to the structuring of public services, functioning as “an administrative technique for rationing scarce resources”.^26p.15^ Rooted in ideology and pragmatism, governments have often deployed stigma discourses to justify cuts to public services and to construct consent around who ‘deserves’ to access services and the quality of care particular groups should receive.^17,27^ The post-2010 UK Coalition and Conservative governments, for example, utilised stigma to justify cuts to Social Security and wider public services.^28^ Once in place, stigma can reduce political opposition to reductions in the quality and coverage of public services supporting those from stigmatised groups, while simultaneously discouraging their uptake.^17,29^

Turning first to the use of stigma processes to keep people *down,* stigma can assist in constructing and legitimising a view of those stigmatised as being inferior in personal attributes, worth, or value.^25,30^ These are actioned through policy and through the shaping of societal attitudes which facilitate the devaluation, domination, and exploitation of those who are stigmatised. In operationalising this, we place focus first on people's experiences of structural stigma processes resulting in downward (material and symbolic) placement in social hierarchies *prior to* accessing the health care system and, following this, how these dynamics are reproduced within this power-laden setting and how it comes to influence their engagement with health care and the quality of care received.

Once kept *down*, people are more susceptible to being kept *in* and/or *away*. Phelan and colleagues^25^ conceptualise keeping people *in* as the use of stigma processes to enforce social norms. Shaming discourses and more formal sanctions are used to punish those who fail to comply, serving to communicate what is deemed (un)acceptable. In our analysis, we focus on how structural stigma processes are used to keep people *in (line)* with explicit and ‘softer’ rules for ways of engaging with health care services, with sanctions dispensed to those not able to present ‘appropriately’.

Keeping people *away* is defined narrowly by Link and colleagues as rooted in the “avoidance of disease . . . rooted in our evolutionary past rather than in current social pressures”,^25p.9^ and later as “a general distaste for deviations standard for the way humans are supposed to look or carry ourselves”.^15p.26^ We argue that the former conceptualisation is too restrictive, and that the latter remains unhelpful in contemporary analyses of structural stigma. Both fail to capture stigma motives across political and institutional contexts. In these contexts, keeping people *away* can instead stem from motives of resource protection, such as the use of stigma discourse and policy to restrict access to public services.^26^ We therefore substitute keeping *away* for keeping *out*, adapting the concept to explore structural and institutional policies, practices and processes that result in the effective exclusion of stigmatised individuals and groups from public services, including health care.

## Missingness in Health Care

Epidemiological work in Scotland demonstrated that people experiencing missingness are more likely to experience socioeconomic deprivation, high levels of multimorbidity, and far worse premature mortality outcomes than patients missing no appointments.^2,31–33^ The phenomenon of *missingness* is defined as “the repeated tendency not to take up offers of care, such that it has a negative impact on the person and their life chances”.^33p.4^ A recent realist review of the literature on multiple missed appointments identified a complex range of interlinking material and psychosocial causes of missingness, as well as several weaknesses in the evidence base.^1^ Foremost, studies typically fail to differentiate between occasional, *situational* missed appointments and chronic patterns of multiple missed appointments that signify more *enduring* barriers to care.

This review, like others,^34^ highlighted a need for a deeper understanding of the contribution of relational determinants such as stigma to missingness, and of their wider structural, political and economic context. Our exploration of structural stigma contributes to this understanding, illuminating how experiences are systematically patterned within and across a range of stigmatised statuses. Doing so shifts away from the predominant ‘individual deficit’ view of missed appointments^1^ towards understanding the deficits of systems and structures that contribute to missingness. In doing so, our intention is not to vilify health care professionals. Importantly, the majority of those who shared their experiences of stigma did not blame individual health care workers but instead recognised the structural and institutional resourcing constraints contributing to these dynamics.

## Methods

### Background Study

This study is part of a programme of research exploring the causes of and interventions to address missingness in health care, undertaken in England, Scotland and Wales in 2022–2024. The data presented in this article are drawn from semi-structured, qualitative interviews with professionals working across health and social care, and experts-by-experience of missingness. Professionals were recruited via email from a range of NHS, governmental, and third-sector organisations, and were often known to research team members through professional links. Experts-by-experience were recruited via gatekeepers across various third-sector organisations using a range of methods: email, text message, online bulletin posts, or in-person at services. While primarily targeting inclusion health populations (‘Inclusion health’ refers to health care approaches targeted at people most negatively affected by health and social inequalities and experiencing acute forms of marginalisation, such as those experiencing homelessness, prison, and/or problem substance use.^35^), participants were sampled purposively to capture data on a wide range of experiences associated with risk of missingness (see Table 1) as well as for heterogeneity in gender and region of residence.

**Table 1.** Characteristics of Interview Participants.

Category	Service/Vulnerability	No. Participants
Professional	Third-sector specialist service pertinent to inclusion health	10^a^
Professional	NHS inclusion health	8
Professional	Local/national (Scotland/United Kingdom) level strategic/policy role	6
Professional	NHS mainstream health care	5
Professional	NHS specialist service pertinent to inclusion health	4
Expert-by-Experience	Participants with experiences conferring risk of missingness: homelessness, asylum system, poverty, domestic abuse, diabetes, learning disability, mental health condition(s), problem substance use, having been in care, or having a caring role.	28
Total		61

a This includes three participants who were professionals with personal lived experience of missingness.

The interviews focused on participants’ perceptions of the causes of missingness, and what interventions might help to address it. The interview schedule allowed substantial flexibility to accommodate focus on each participant's area of expertise. The interviews were undertaken by co-author DB between April 2023 and July 2024, either in person at various locations (eg, University of Glasgow offices, cafes, and a drop-in homelessness centre) or online via Microsoft Teams or Zoom.

## Analysis

The interview recordings were transcribed verbatim and the data analysed by DB using NVivo 14. Analysis was undertaken abductively, and was informed by a theoretical framework, detailed elsewhere^1,36,37^,centring on fundamental cause theory. Fundamental cause theory (FCT) sees socioeconomic position and, in more recent formulations of the theory, race, power, and stigma as fundamental causes of health.^6,22,30^ Epidemiological evidence demonstrates that health inequalities have persisted across time and place, and throughout sweeping changes in intervening mechanisms. FCT suggests that this is because these fundamental causes shape durable, generative *metamechanisms* which account for the generation and replacement over time of intervening *mechanisms* (specific causal links between fundamental causes and health outcomes in any given context) in such a way that these mechanisms, in sum, sustain the overall causal relationship. Theorised metamechanisms include, among others, unequally distributed flexible resources (such as knowledge, money, status, and beneficial social connections) and institutional processing (the unequal treatment of individuals and groups at the institutional level). Relevant to this article's focus on structural stigma, FCT suggests that policies targeted at intervening mechanisms are insufficient to address health inequalities, and addressing health inequalities requires structural policies targeted at these fundamental causes.^22,30,38^ An initial coding frame was developed, consisting of five levels: structural (economic and political structures), living and working conditions, institutional, community and social networks, and individual-level determinants. Mechanisms risking missingness were coded at each level. The selection of mechanisms was informed by, triangulated with, and used to refine our emergent theory about missingness drivers from the realist review of the existing literature.^1^ Stigma was coded at each level where relevant. Its foregrounding here stems from having frequently been cited by professional and expert-by-experience participants as a prominent driver of missingness, and from its relative absence from existing research on missed appointments.

Coding and analysis were discussed and refined through weekly ‘data surgeries’ involving co-authors DB and MM and discussed in team meetings with all authors. DB, who undertook all interviews and analysis, was an ‘outsider’ to both sample groups. However, reflexive work was aided by ongoing discussions with the multidisciplinary research team (which included research and professional expertise in inclusion health and health inequalities), our public co-investigator, and our stakeholder advisory group, who provided extensive feedback on our methodological and analytical orientation.

### Limitations

Sample size limitations prevented sampling from all stigmatised statuses potentially conferring risk of missingness. Nor have we fully captured all structural stigma processes affecting specific groups. However, our intention is not to generalise to the experience of specific stigmatised statuses but to understand structural stigma processes as they cut across diverse patient groups.

Additionally, placing focus on a single outcome—missingness in health care—may downplay the role of structural stigma in affecting our participants’ lives. Structural stigma likely affects experiences across a wider range of services, social networks and opportunities, as well as contributing to behavioural and psychosocial pathways to poor health.^21^

## Findings

Across professionals and experts-by-experience, stigma was foregrounded as a prominent mechanism structuring access to, and experiences of, health care settings. We present findings below in relation to our three stigma outcomes: *keeping people down*, *in (line)*, and *out*. Across these categories, we explore findings relating to three case-study stigmatised statuses: marginalised racial and ethnic identities (including seeking or awaiting asylum processing), mental health conditions, and problem substance use. It is important to reiterate that the structural stigma processes identified were not limited to these three statuses. Further, we acknowledge that these categories are heterogenous and intersect with other axes of inequality, including social class and gender.

### Keeping People Down

Mechanisms through which structural stigma processes served to keep people *down* were apparent across all three stigmatised statuses. Here, we explore the impact of structural policies pertinent to interviewees’ socioeconomic position and status, processes of ‘scapegoating’ by those in positions of power, and instances of being treated as ‘less than’ within health care.

#### Social Policies Keeping People Down

Reflecting the notion that structural stigma extends far beyond “one person do[ing] something bad to another”,^11p.382^, interviewees across these three stigmatised statuses spoke of their experiences of structural policies designed to keep groups of people down. The intricate relationship between structural stigma, socioeconomic position, and missingness was most apparent in the accounts of those with experience of the asylum system. These participants spoke of the insufficiency of income provided for those in the asylum system, and its impact on their ability to sustain access to health care and other essential resources:

“They were giving me a £30 [weekly] stipend . . . . They said that I needed to check into the hospital every single day . . . . Most times, really, I missed the appointments, because I just couldn’t afford to get myself there. I had to choose between food and go[ing to] hospital.”

(Anna, EBE4)

These challenges were compounded by policies prohibiting employment for those in the asylum system, which participants described as consigning them to poverty and negatively impacting their mental health. Those whose claims had been approved faced homelessness and precarious employment:^39^

“If you get leave to remain as an asylum seeker, you automatically enter the homelessness system.”

(Justine, Pro15)

“We have a lot of doctors who just become Uber drivers . . . . They studied for more than seven years, and after that they just go to some restaurants and mop the floor.”

(Fatima, EBE22)

Policies keeping people with mental health conditions and problem substance use *down* were also apparent. With those living with more severe mental health conditions and problem substance-use often facing barriers to employment,^40^ participants spoke of the negative effects of the increased conditionality of the Social Security system under austerity:^24^

“We had somebody in yesterday . . . with quite complex physical and mental health problems but [who] had managed to get a very part-time job . . . . That person was told they needed to work more to get off Universal Credit (Universal Credit is a Social Security payment for those of working age who are on low income in the United Kingdom. It covers those who are looking for work, or who are employed but receive a low income). This person had . . . managed to achieve a sort-of status quo [balancing their health and employment needs]. And then that pressure from the benefits system . . . which is trying to get people off every tier of benefits . . . had made that person ill.”

(Ruth, Pro23)

#### Scapegoating: Positioning as Less Deserving of Public Services

Participants suggested that the downward placement of groups such as ethnic minorities and migrants has once again become pervasive across the United Kingdom in the context of austerity and Brexit, with these groups constructed as ‘scapegoats’. Professional and expert-by-experience participants frequently expressed awareness of the sociopolitical context within which stigmatisation is amplified, and of how stigma discourses are explicitly used to justify the kinds of structural policies outlined above:^16,39,41^

“I’m really actually genuinely worried that we’re in a political cycle that's blaming the other person for things that are systemic . . . . It's definitely not true that asylum seekers are the government's reason why everything is failing.”

(Justine, Pro15)

Several expert-by-experience participants highlighted their acute awareness of narratives constructing them as less ‘deserving’ of public services, including health care. Pinker^26^ highlighted that the roots of stigma associated with the use of public services relates to the extent that individuals and groups are seen to be ‘dependent on’ or ‘contributing to’ public services over the lifecourse. Participants with mental health conditions and other forms of disability spoke of having become cast by politicians and media as a burden on public finances as a means of justifying austerity. Jenny (EBE27) described herself as “filled with anxiety at the moment because the government are making real scapegoats out of disabled people”. Referencing increasingly conditional UK government Social Security policies of the 2010s to early 2020s, Vincent, who lives with mental health conditions and neurodiversity, had been made to feel that:

“I’ve become a drain . . . . Politically, you see the kicking and the drubbing that disabled people get when the economy turns . . . . The first thing they came out with . . . was, ‘oh we’re going to put more sanctions’. (Sanctions are a punitive component of conditionality in the UK Social Security system, whereby payments of Universal Credit, Employment and Support Allowance and Jobseeker's Allowance can be reduced or withdrawn for perceived noncompliance in several conditions).”

(Vincent, EBE 14)

All participants who had experienced problem substance use described feeling negatively perceived by wider society and health care services. These participants often identified this as stemming from narratives about responsibilisation, which blames individuals and undermines the legitimacy of support.^42^ These dynamics appeared particularly acute among women, stemming from gendered understandings about responsibility:^43,44^

“There's a lot more guilt and shame for women, full-stop, being addicts . . . . We, as a society, we have the worthy sick and the unworthy sick . . . . We don’t fund this [substance use recovery services] very well . . . . So, what does that tell people? It just says ‘it's your fault. So, why should you expect good quality healthcare?’”

(Sharon, Pro-EBE3)

Some participants spoke of having internalised discourses constructing them as undeserving of health care.^45^ Jim's account highlighted how this compounded his already low self-esteem, which he attributed to childhood poverty and parental problem substance use:

“The feelings ae inadequacy and ‘less than’ probably override everything else . . . . When . . . you see yourself as one of the least deserving people, when somebody reaches their haund and . . . even in this instance where it's engagement wi a service . . . because you believe already that you don’t deserve it, you arenae gonnae take the haun’.”

(Jim, Pro-EBE2)

#### Being Treated as ‘Less Than’ Within Health Care

As well as being constructed as ‘less than’ in wider political and media discourse, participants regularly described the ways in which these stigma processes also operated to keep them down within the health care system. As in previous research,^46^ those from racialised minorities often felt they received inferior treatment, while finding discrimination challenging to prove:

“I was the first one to get there [walk-in clinic]. For some reason, I left the last person. And there was a lot of white ladies and they left. And even when it was my time, there was supposed to be at least four [members of staff] there. There was just one person tending to me. And I felt like, ‘well I saw the way you guys attended to the other ladies, why has my one changed?”

(Allison, EBE5)

People across all three statuses spoke of the power of labelling and the downward placement emanating from this.^11,47^ People often felt judged negatively by health care professionals, and that they were not listened to or taken seriously. Those who had experienced mental health conditions and problem substance use often described feeling that they were reduced to being seen only as their ‘symptom’:

“I’m not a nameless face, oh ‘another alkie’, ‘another junkie’ . . . . So often when you go into services you are your issue. You’re not a person . . . . My theory is the more poorly resourced services are, the less time they have to treat you as a human.”

(Sharon, Pro-EBE3)

Some accounts provided clear evidence of societal narratives of ‘undeservingness’^39^ being replicated within health care settings by professionals. These were most prevalent in relation to problem substance use, with ‘othering’ and devaluation of people drawing from wider discourses about responsibilisation:

“I took an overdose [. . . and was] waiting to go upstairs onto the ward. And there's two nurses with me and one of them said to the other one, ‘I don’t see why we should waste NHS money on scum like that’ . . . . Every time I go to hospital, that's the memory that comes back.”

(Alex, EBE6)

As one general practitioner (GP) suggested, the “systemic problem” of collective or personal experiences of structural stigma over time contributed to collective mistrust in health care across stigmatised groups, resulting in people “feeling disengaged” and “choosing not to” attend (Anika, Pro8). Having been apparent in ‘vaccine hesitancy’ among some minoritised ethnic communities,^48^ participants’ accounts at times reflected a wider alienation and lack of trust in the health care system to address their needs in a way that keeps them physically and psychologically safe.

Across these participants, the cumulative nature of structural stigma processes keeping people *down* contributed to a reduction of power and agency in relation to their ability to present and advocate for care. While primarily serving to ‘clear the ground’ for keeping people *in (line)* and *out*, these processes were often enough alone to compel people to disengage from health care services.

### Keeping People in (Line)

The interviews highlighted a range of stigma processes enacted within health care settings aimed at keeping people *in (line)* with the forms of presentation aligned with the effective functioning of a universal, free-at-the-point-of-use health care system. Interviewees highlighted that these requirements often failed to interface with the life circumstances and/or clinical symptoms of those interviewed. Those falling outwith these bounds often faced sanctions, resulting in them experiencing services as challenging to access and resulting in a feeling among some participants that “we’ve been set up to fail” (Vincent, EBE14). Here, we explore the inflexibility of health care services and systems to ‘alternative’ presentations, and poor practice regarding reasonable adjustments for those from these three stigmatised statuses who faced challenges to attendance.

#### System Inflexibility to Alternative Presentations

The inflexibility of systems to accommodate presentations not in keeping with the needs of the health care system often meant poorer care for those interviewed. Several professionals highlighted that ‘rules’ for access and engagement with health care are aligned to those who have sufficient material and cognitive resources, and who were well enough to present in a manner aligned with service needs (eg, to maintain a perfect attendance record; to present politely, on time, and with a hygienic appearance; to trust and engage smoothly with professionals; and to adhere to prescribed treatment). This was the case even across specialist health care services targeted at those likely to face such challenges:

“[We’re] a [psychological] trauma service and we expect people to come along, climb some stairs, sit in the waiting room, . . . meet someone they’ve never met before, go into a room, have the door shut behind them and then start talking about some stuff. And not everyone can do that . . . . We’re seeing the people who turn up and who are relatively easy to treat.”

(Keith, Pro24)

These barriers to access and engagement in health care included both relational and material challenges. Our findings highlighted a lack of system understanding of, or flexibility to, accommodate these challenges, rooted in a tendency to see them as ‘individual failings’ contributing to individuals’ ‘lack of engagement’:

“Our local hospitals have introduced an app, and if you don’t use their app, then you can’t really use their service . . . . And now they just allocate you an appointment anywhere, really, and it can be the other side of the city. And it seems to be designed by middle-class people with transport and money, and organisational skills, for a population that doesn’t have those things.”

(Justine, Pro15)

Similarly, participants suggested that service inflexibility made accessing care particularly challenging for those experiencing problem substance use—particularly around abstinence protocols. Compounded by long waiting times in accident and emergency (and other) services, people with physical dependency on substances would fear developing withdrawal symptoms:

“[It's] a reason for leaving the hospital, and missing treatments, essentially coming to harm because their addictions are not managed adequately . . . . I don’t really know who we’re treating with all these diktats, but certainly not the patients.”

(Nicola, Pro26)

#### Poor Provision of ‘Reasonable Adjustments’ for Support to Attend

Link and Hatzenbuehler^49^ highlight the importance of policy inaction in enacting structural stigma. This was apparent throughout our interviews in the form of poor provision of necessary additional support to attend and engage with appointments. Those from racial and ethnic minorities who faced language barriers to engagement described inconsistent provision, with NHS translation services often poorly utilised, and not always available:

“I was like, ‘what do you need?’ . . . She was just trying to tell them that she's in pain. And she felt that her waters [were] leaking, but she couldn’t say it in English . . . . They don’t have the time to actually assess her or communicate with her.”

(Allison, EBE5)

Having been involved with services for decades, Vincent spoke of the withdrawal of the reasonable adjustments previously granted to him to support his effective engagement in appointments. While a legal requirement under the Equality Act 2010,^50^ this withdrawal was felt by some participants to reflect the lack of parity given to mental health conditions and relational disorders. Vincent linked this to cuts to the resourcing of UK services across the previous decade:

“The systems are collapsing . . . . My conditions, diagnoses have never changed, they’ve all told me. However, the rationing has begun . . . . People, service providers, on the ground have become ragged . . . . They certainly didn’t go into it to make people iller, and I’m not suggesting they are, I’m suggesting that the system is . . . . They said they’re doing away with double appointments . . . . I’m not being funny, but it would be like not having accessible toilets.”

(Vincent, EBE14)

For those facing such challenges, these often cumulative barriers resulted in poorer access to and quality of care, and, often, disengagement. Keith, a psychologist, described how this pattern plays out among those at his service:

“Both sides often fall into the re-enactment of the history where the care provider's like, ‘we don’t want to see that person again because they’re unpleasant’, and the care seeker is like, ‘see, I told you, care providers are full of shit, they don’t really want to see you anyway and they always say that you’re the problem’.”

(Keith, Pro24)

Link and Phelan's study of people with mental health conditions^15^ demonstrated how the aims of those enacting stigma were achieved through people's anxieties around *keeping in (line)* with normative boundaries. While we heard of such concerns, our findings also foreground the challenges faced in adhering to these formal and tacit rules governing service engagement. While these may be said to be ‘successful’ in the eyes of those enacting stigma in that they worked to maintain order and efficiency in an under-resourced health care system, they also served to reduce people's confidence in the health care system, and/or encouraged people to *keep out* through withdrawal from care.

### Keeping People out

Participants described various stigma processes serving to keep people with these stigmatised statuses *out* of health care settings. In their study of stigma affecting people living with mental health conditions, Link and Phelan^15^ proposed that when efforts to keep people *in* fail, practices to keep people *away* are deployed. Our findings, however, include mechanisms keeping people *out* which were deployed prior to allowing people the opportunity to keep *in (line)*. We present findings below in relation to exclusionary ‘gatekeeping’ practices, the ‘rationing’ of care to groups posited as less ‘deserving’, and more subtle processes of disengagement over time with patients perceived as more ‘challenging’.

#### Gatekeeping Practices

Our interviews highlighted how, when attempting to register and arrange appointments in primary care, people experienced exclusionary ‘gatekeeping’ practices which served to keep them out. While GP surgeries in the United Kingdom are not legally allowed to refuse to register people without identification or proof of address, our findings suggest that this is a common practice, and it has the consequence of excluding a large proportion of those from asylum backgrounds or experiencing homelessness, and who are likely to be unaware of legislation, or unsuccessful in advocation:^51^

“What I keep hearing is that . . . they might be desperate for medication, desperate to see a GP, but the receptionist is saying, well, I can’t register you because you haven’t got ID.”

(Adam, Pro9)

One GP reported that patients in the asylum system regularly came to register at his practice having been sent there by other practices. While his practice does offer support tailored towards immigrant populations, he suggested that this was also driven by a discriminatory hesitancy to register people deemed to be part of a “difficult population”, both in terms of clinical presentations (high rates of post-traumatic stress disorder, for example) and in terms of the extra support required (including language support):

“When they have approached other practices nearby they’ve been told, ‘okay, no, we are closed, go to Lloyd's practice, they take new patients and they are taking asylum seekers’. . . . They were not being encouraged to get registered.”

(Lloyd, Pro20)

These challenges have been exacerbated by the withdrawal of payments to help practices cope with the extra support often required, risking the ‘ghettoisation’ of those in the asylum system into services burdened by high and complex caseloads and low resources.

Alongside more subtle, challenging-to-prove stigma processes, some participants spoke of instances of direct racist discrimination when attempting to register for health care. Niamh, a support worker for a Gypsy Traveller advocacy organisation, described supporting a woman experiencing domestic abuse in registering at her local GP:

“‘Can I just ask your name?’ . . . . ‘McGinley’. And she said, ‘I’m afraid we’re not taking any more McGinleys on the books, we’ve already got too many of them’ . . . . You talk to people and they’ll say ‘oh God, yeah, that's happened to me loads of times’.”

(Niamh, Pro18)

#### Rationing of Health Care Services and Wider Support

When not fully excluded, the ‘rationing’ of offers of medical treatment or support served as a further mechanism keeping people out of services offered to the wider population. This was particularly apparent for those with prior or ongoing experience of problem substance use. Practitioners across inclusion health services had supported those denied treatment from mainstream NHS services, at times attributing this exclusion to the greater stigma stemming from the fact that problem substance use affects more people towards the lower end of the socioeconomic gradient:

“If someone comes in with a serious heart infection from injecting drug use . . . they will be denied life-saving interventions, frequently, I mean, as standard . . . . A heart valve, a metal heart valve, is not such a limited resource. And there is no attempt made, psychologically, psychiatrically, in any way, to see whether this patient has a chance.”

(Natalie, Pro17)

Similar withdrawals of care extended to medical interventions which would not be contradicted or carry increased risk of complications through prior or ongoing substance use:

“We have a female patient who's had a leg amputation . . . . And she hadn’t been referred for any physiotherapy follow-up to deal with the amputation, and to work on her mobility, and work towards getting a prosthetic limb . . . . They said, ‘well we haven’t referred her to physio because she has ongoing issues with IV drug use’ . . . . It's exactly the kind of thing we come across all the time . . . .”

(Louise, Pro3)

While ongoing substance use (or interrupted ‘recovery journeys’) should not jeopardise patients’ legitimacy as candidates for health care, some interviewees argued that stigma processes were rooted in negative valuations of people experiencing problem substance use as ‘not worth treating’. Jason—incidentally, now an advocate working towards UK government drug reform—spoke of his history of crack cocaine use, and his perception that he had been ‘written off’ by the NHS following a suicide attempt:

“The NHS attitude with me was, because I was a drug addict, a black, male, homeless drug addict, there was no point in the NHS spending any money on my rehabilitation. Because I was just gonna go back to doing drugs again. The usual.”

(Jason, EBE16)

This rationing of care across these groups wasn’t apparent only in relation to medical procedures, but to wider offerings of support to attend appointments. One professional questioned why ambulatory support was not offered to people experiencing problem substance use:

“My feeling is that . . . there's a judgement made. Certainly around the drug-using population, there's a definite judgment made that they’re not . . . or any kind of addiction, alcohol, any addiction-affected individual, is not offered any of these services. Because there's a feeling that they will not attend, I think.”

(Nicola, Pro26)

#### Exclusionary Responses to People with Complex Social and Clinical Needs

While directly exclusionary processes were evident, the findings also highlighted the cumulative effect of often small and subtle structural stigma processes in keeping out those perceived as more ‘challenging’. Those who had formerly worked in mainstream health care settings suggested that health care professionals tended to, often unintentionally, prioritise patients who fitted an ‘ideal’ type, and, over time, served to exclude (often through subtle processes of non-engagement such as not following up with ‘missing’ patients) those from stigmatised populations who they tacitly felt to be seen as less ‘treatable’ and relatable. These patients were often those with a high burden of complex social and medical needs:

“A 44-year-old man with a history of schizophrenia . . . . We tried to work with community nurses and diabetic specialist nurses . . . . There was, sort of, a great deal of therapeutic nihilism about . . . ‘well you can’t work with people like that, or what do you do if they’ve got multiple social problems? It's not something that we can do anything about.’”

(Ruth, Pro23)

Several participants highlighted that the non-evoking of sympathy for certain clinical and social challenges—typically those seen as rooted in personal ‘choice’, and therefore blameworthy—was exacerbated by social distance between professionals and patients, with professionals rarely having been exposed to the lived realities of those experiencing these vulnerabilities:

“Nearly every doctor's surgery has got like, their ‘who we are’ page, and that shows you how much they earn each year . . . . And fair dos, you know, they’ve put the work in . . . . But I think they’re just distanced from like what is really going on . . . . So, it's all, it's like a blame culture, and ‘if people don’t want to help themselves, why should we go out our way to help them?’”

(John, EBE7)

Indeed, talking about the risk of self-discharge in such patients, one professional (who distanced herself from such perceptions) suggested that there exists a sense across services that:

“[I]t's almost solving a problem, isn’t it, if that patient is, you know, no longer there?” (Louise, Pro3).

While we previously saw that people were often effectively kept *out* of services by stigma processes which also kept them *down* and *in (line),* our findings have demonstrated more directly the ways through which structural stigma processes keeping people *out* of health care services were achieved. Accounts from all three case studies highlighted directly exclusionary policies and practices apparent across health care services, resultant *intrapersonal* stigma through self-exclusion and withdrawal from care in the face of potentially threatening interactions, and the subtle processes through which patients were ‘disengaged with’ over time. While arguably “solving a problem” for resource-constrained services, these processes are likely generating negative outcomes for patients given the mortality outcomes observed among those experiencing missingness,^29^ and the likely far greater risk faced by those who become missing from health care entirely.

## Discussion

While a wide range of material and psychosocial pathways and mechanisms can contribute to missingness from health care,^1^ our findings have demonstrated that stigma plays a central role across these three case-study statuses. These findings provide further evidence that stigma extends far beyond the predominant *interpersonal* model towards being rooted and implicated in wider societal processes and policies. While having placed focus on three stigmatised statuses, we demonstrate the ways in which structural stigma processes worked across these groups to shape access to and the quality of care, emphasising the need for a universal approach to understanding and addressing stigma. While this study has focused on interviews relating to the health care system in the United Kingdom, the processes identified are likely widely applicable beyond this country and across other public services.

Our findings have demonstrated the salience of the concepts of keeping *down*, keeping *in (line)*, and keeping *out* for exploration of structural stigma mechanisms. We show how both structural policies and discourse—deployed by those in positions of power—acted to devalue and dominate those across these three case studies within and outside of health care settings. Structural policies associated with the asylum and benefits system reduced people's power and agency, including in relation to health care engagement. While often resisted by participants, we saw that structural discourses placing people *down* were influential in shaping processes of *interpersonal* stigma, as well as *intrapersonal* stigma, in that they were often internalised by those who were stigmatised and resulted in a hesitancy to present to health care settings. Those who did present to services faced a rigid system of formal and informal rules designed to keep people *in (line)*, with soft or formal sanctions for those who did not present in a manner in keeping with the present functioning of the health care system. Our findings demonstrate the substantial barriers to engagement that this caused for many of our interviewees (reflecting research highlighting the significant ‘patient work’ required to mitigate ‘anticipated and received’ stigma.^52^ Further, we found a range of processes through which our participants were kept *out* and excluded from health care treatments or services, with these achieved by formal or soft policies either intentionally or unintentionally enacted by health care services and professionals, or through the withdrawal and exclusion associated with missingness.

Link and Phelan^15^ use the concept of stigma power to denote the subtle and often ‘misrecognised’^53^ processes through which stigma processes achieve the aims of powerful actors for whom stigma is an effective tool. While these were apparent throughout our study, our findings also demonstrated a range of less subtle structural stigma mechanisms keeping people *down, in (line)* and *out* across health care settings. The persistence of, and relative lack of policy attention to, these structural stigma processes may be said to stem from structural stigma processes keeping people *down* symbolically and materially outwith the health system, and the reduction in individual and collective advocacy which results from this. We therefore suggest that Link and Phelan's concept of ‘stigma power’ represents one dimension of a wider array of stigma processes.

In seeking to understand structural stigma in this context, we return to Tyler's assertion that if stigma did not benefit someone, it would not exist,^17^ and Pinker's proposition that stigma forms a powerful administrative tool for the distribution of scarce public resources.^26^ While not explicated, stigma was felt by several participants to have been purposively deployed by those in political positions as a means through which to ration statutory services, and to construct and amplify notions of who is ‘deserving’ and ‘undeserving’.^17,54^ The post-2010 UK Coalition and Conservative governments’ drive to generate a ‘hostile environment’ for immigrants to the United Kingdom without legal leave to remain in effect ‘deputised’ these policies to employers and those working across public services.^55^ Findings here suggest similar processes in response to real-term cuts to NHS funding under austerity. Increasingly flanked by a sharpening of political and media discourse around ‘deservingness’ of health care services, health care planners are compelled to make decisions regarding allocation of a scarcer pool of resources as a necessity of managing these cuts.

While some have questioned the assumption that “stigmatisation always represents a threat to public health”^56p.3^ in that it might encourage behaviours which are health-promoting—arguments countered by recent empirical research^57^—our findings demonstrate that stigma forms a blunt and “barbaric tool”^58^^,p.474^ which “in health facilities is particularly egregious”.^59^^,p.1^ Our findings demonstrate that it is not only ineffective but counterintuitive in preventing stigmatised people from engaging in health-risking behaviours,^60^ but risks substantial harm to patients through contributing to health care disengagement.

Like other studies employing the lens of structural stigma,^3,15,49^ the findings presented here suggest what types of policies and interventions stand a better chance of addressing stigma, and which are likely to be ineffective. Public services, as an important interface between statutory drivers, communities and individuals, can play a vital role in buffering against these wider stigmatising processes. However, stigma relating to health care and other public services is only one domain through which stigma affects the lives and life chances of people who are stigmatised.^3^ While pragmatic in the short term and often of great importance to individuals’ lives, interventions targeted at the level of the individual are unlikely to result in a sustained decrease in structural stigma processes and leave untouched an array of mechanisms through which it generates negative outcomes.^9–17^ The concept of structural stigma foregrounds the notion that, while powerful actors are motivated to enact stigma to achieve various ends, either these motivations or the power to enact these processes must be addressed: “otherwise, social and health inequality will be reproduced”.^3^^,p.817^ Indeed, several interviewees, including a civil servant, highlighted a lack of ‘political will’ at this level in addressing health inequalities.

A recurrent theme throughout our findings was the context of austerity and its attendant severe resource constraints both outwith and within the health care system, which generated the conditions for the exacerbation of stigma processes. These findings point to both an increase in funding for mainstream health care, and for targeted funding—under the principle of proportionate universalism^61^—to provide the additional support required for people from across a range of stigmatised conditions and with structural vulnerabilities to access and engage with health care (we have expanded more fully on a range of interventions to address missingness in another article.^36^). Without a wider uplift in funding, our findings suggest that stigma may play a role in challenging such redistributive aims. As argued previously,^29^ there exists a double bind; policy inaction perpetuates inequalities in access, while policy action—without prior or concurrent action on stigma—not only risks reducing public support for such support but risks aggravating the stigma attached to those who fall under the banner of ‘inclusion health’. Further, while it is essential to avoid reproducing stigma within health care settings, and while they can form an important arena through which to counter wider processes, interventions at the level of health care settings alone will fail to address stigma.^3,11^

## Conclusion

Focusing on missingness in health care, our findings have demonstrated the importance of extending beyond individualist conceptualisations of stigma towards exploration of structural stigma and its impact on people's experiences and outcomes across a range of domains. In doing so, we suggest a focus on the ways in which stigma is produced by those with greater power, the ends it seeks to serve, the processes through which this problem ‘trickles down’, and the lived experience of these processes among those who are stigmatised. While a universal theory of stigma applicable across different contexts is likely not imminent, future research should continue to move beyond the personal tragedy convention^6^ towards rigorous exploration of the structural mechanisms resulting in poorer outcomes across a range of domains across a range of stigmatised identities. In doing so, attention should be paid to the function which stigma plays across different sociopolitical contexts and across different social and institutional settings.^25^ This article has made a substantial contribution to such efforts in elucidating and refining the concepts of keeping *down, in (line)* and *out*, and demonstrating their utility for analysis of structural stigma processes as they come to shape inequalities in people's experiences and outcomes across health care settings.
